# Implementation of medication reviews in community pharmacy: reaching consensus on stakeholders’ recommendations for mechanisms of change using the nominal group technique

**DOI:** 10.1007/s11096-024-01708-y

**Published:** 2024-03-15

**Authors:** Dorothee E. Michel, Antonella P. Tonna, Dorothee C. Dartsch, Anita E. Weidmann

**Affiliations:** 1 School of Pharmacy and Life Sciences, Robert Gordon University, Garthdee Road, Aberdeen, AB10 7GJ Scotland; 2 Cap Campus Pharmazie GmbH, Planckstraße 13, 22765 Hamburg, Germany; 3 Department of Clinical Pharmacy, Innsbruck University, Innrain 80, 6020 Innsbruck, Austria

**Keywords:** Community pharmacy services, Implementation, Mechanism of change, Medication review, Nominal group technique

## Abstract

**Background:**

Since 2022, patients with five or more medicines are eligible for a medication review (MR) in a community pharmacy remunerated by the German health system. However, implementation has been slow, with few pharmacies providing MRs. Stakeholders’ input is necessary to detail how implementation strategies can be executed effectively on a national level. Prior research identified “external facilitation” and “altering incentives” as crucial strategies to achieve implementation outcomes.

**Aim:**

To gather stakeholders’ recommendations for, and obtain consensus on, mechanisms of change that allow implementation strategies to work in practice.

**Method:**

The consensus method used was the nominal group technique (NGT) with NGT-discussions held separately with pharmacy owners and pharmacy chambers employees. Votes were summed and the relative importance (rI) calculated, defined as (score achieved for a mechanism)/(maximum possible score) × 100. Content analysis provided context for the highest ranked mechanisms and allowed linking to implementation outcomes.

**Results:**

Four NGT-discussions were held in 2023 (n = 2 owners; n = 2 chamber employees) with a total of 17 participants. The overall highest ranked mechanisms were fit-for-purpose software (rI = 154.7) detailed process support (rI = 104.9) and an expert support line (rI = 77.7). These together with financial viability (rI = 40.0) were prioritised by both participant groups. Three mechanisms were favoured for both implementation strategies, namely software, process support and materials (rI = 34.3).

**Conclusion:**

This study identified stakeholders’ priorities for mechanisms of change to implement MRs in community pharmacies. Focusing efforts on the prioritised mechanisms is likely to significantly advance a national implementation plan for countries which are at an early implementation stage.

**Supplementary Information:**

The online version contains supplementary material available at 10.1007/s11096-024-01708-y.

## Impact statements

- This theory-driven study presents a model for the implementation of medication reviews in a community pharmacy setting.
- Prioritised mechanisms of change were deemed to be effective to improve particularly adoption, appropriateness, and feasibility of the medication review service.
- This study’s findings are suited to inform a national implementation plan for any country considering the implementation of medication reviews.

## Introduction

Medication reviews (MRs) are increasing in importance due to ageing populations worldwide [1]. The prevalence of polypharmacy (defined by Varghese et al. as five or more medications) among the elderly living in the community, averages 30% across European countries [2, 3]. Polypharmacy can cause harm if the medication is not optimised and in fact, there are annually 8.6 million unplanned hospital admissions in Europe due to adverse drug events [1, 4]. To address these issues, community pharmacy practice is increasing in scope to meet modern health care demands, providing services such as medication reviews (MRs) [5, 6] which have been shown to be both cost-effective [7] and to reduce hospital (re-) admissions [8].

Since 2020, patients with five or more medicines are entitled to receive a medication review (MR) in German community pharmacies, aiming to optimise medicines use and improving health outcomes, with MRs remunerated by all health insurances [9–11]. The paid MR-service model aligns with the Pharmaceutical Care Network Europe’s definition of an MR type 2a i.e. using a patient’s medication history and a patient interview as sources of information [10]. Despite remuneration and an official mandate recommending provision of MRs, the implementation by German community pharmacies is slow [12]. For MRs to have a meaningful contribution to patient safety and to support optimisation of public health outcomes the widespread implementation of MRs is necessary. However, implementing new evidence-based services into practice settings faces some well-documented challenges such as lack of staff, providers’ resistance to change, patient lack of awareness of services, and general lack of resources and training [13–17]. Implementing MRs into a community pharmacy setting is no exception as highlighted by a systematic review [18]. While many employed pharmacists were willing to provide MRs, they complained about generally lacking support from pharmacy owners, so that the owners appeared to be drivers of change or rather hindering change in this case [19]. Implementation science suggests starting any strategic implementation plan with the identification of barriers and facilitators in the respective context, followed by the development of strategies to address the barriers and leverage facilitators [20]. As implementation strategies can be complex, stakeholders’ input is crucial to identify mechanisms of change which are likely to facilitate the implementation of MRs in the practice setting and to achieve implementation outcomes [20, 21]. An implementation outcome can be regarded as a short-term or intermediate milestone which needs to be achieved on the path to improved health outcomes. Poor implementation of an intervention impedes reaching its full effect on a large scale [20, 22].

The German health care system is based on the Bismarck model where users pay a fee that is reinvested into the health system; health insurance is mandatory and is mainly provided through Statutory Health Insurances [23]. All German community pharmacies are privately owned by registered pharmacists and third-party ownership is not allowed [24]. As only dispensing of medications is mandatory, pharmacy owners are free to decide which additional services they offer. Across Germany, 17 regional chambers deal with the professional interests of all registered pharmacists [25]. This includes supporting the provision of continuing professional education which enables practicing pharmacists to deliver up-to-date care. Prior to the recent commission of MRs (along with other clinical services), the remuneration of community pharmacy was based solely on dispensing activities [26]. Medication reviews had been performed on a small scale within regional projects which provided a first insight into factors that may potentially influence implementation [19, 27]. An interview study with German pharmacy owners identified many barriers and facilitators to the implementation of remunerated MRs and yielded suggestions for implementation strategies [28]. Besides obvious strategies such as financially sound remuneration and strategies outside stakeholders’ influence such as legal frameworks, key suggestions for workable strategies included employing external facilitation to support the pharmacy while undergoing implementation (*external facilitation*) and providing an additional incentive (*alter incentive structure*) to facilitate implementation of MRs [29]. However, the mechanisms of change through which these implementation strategies could target outcomes needed further detail.

### Aim

This study aimed to gather stakeholders’ recommendations for, and obtain consensus on, mechanisms of change that would potentially allow two promising implementation strategies, namely *external facilitation* and *alter incentives,* to work in practice.

### Ethics approval

This study received ethical approval at Robert Gordon University, Aberdeen, Scotland (S323) and a waiver from Hamburger Ärztekammer, Hamburg, Germany (2023-300282-WF). Participants gave written consent prior to commencing data collection.

## Method

### Study design

A nominal group technique (NGT) was used [30, 31]. NGT is a consensus method which allows key stakeholders to generate potential solutions to a given problem and establish consensus [32]. The method has been widely used in implementation research and has the potential to identify mechanisms of change for complex implementation questions [32–35]. As key stakeholders generate the mechanisms themselves, these are more likely to meet their needs and to strengthen ownership of the results as opposed to a Delphi method which involves more input from the research team [36].

### Recruitment

To encourage recruitment, an article was published in a national professional journal highlighting the study background and aim [37]. In addition, recruitment emails with the same information were sent to all 17 regional pharmacy chambers across Germany. Both contained a link for respondents who were interested in participating to complete their contact details, demographic information and to select preferred dates for the NGT online discussions.

### Nominal Group Technique (NGT)

When planning an NGT study, several decisions need to be taken in advance [38]. Scope and number of questions, size and number of groups, and diversity of participants within a group can vary. For this study homogenous groups were used to allow participants the safety of sharing ideas only with their peers. Answering broader questions requires more or larger groups, similar to other types of qualitative research [39]. Since this study focused exclusively on two specific implementation strategies, it was expected that a small number of participants would be sufficient. Data saturation is inherent in the round robin step as naming ideas continues until no new ideas emerge, with the aim of the NGT being to prioritise the most promising ideas with only those ideas taken forward which receive the most votes. Separate NGT group discussions were held for each of the participant groups (pharmacy owners, and pharmacists employed on a full-time basis at regional pharmacy chambers). All NGT-discussions were held online, using Zoom® software (vs 5.11.11). Written consent was obtained from all participants prior to the online NGT-discussions. The NGT started with a brief introduction by the principal researcher (DM) to the research aim and the method. Two questions were posed in each NGT-discussion (Table 1).

**Table 1:** Questions posed in the online NGT discussions

Chamber employees’ NGT-discussions:	Pharmacy owners’ NGT-discussions:
1. *“How can external facilitators support pharmacists to…*	1. *“How can external facilitators support you and your team to…*
2. *“Which incentives would help pharmacists to…*	2. *“Which incentives would help you and your team to…*
*make MRs more acceptable?*	
*adopt MRs in their / your pharmacy?*	
*reduce costs of MR-delivery and sustainment?*	
*make MRs more appropriate?*	
*increase MRs’ feasibility?*	
*deliver MRs with high fidelity?*	
*provide MRs to more patients (penetration)?*	
*sustain MR-delivery over a long period?”*	

**Fig. 1:**
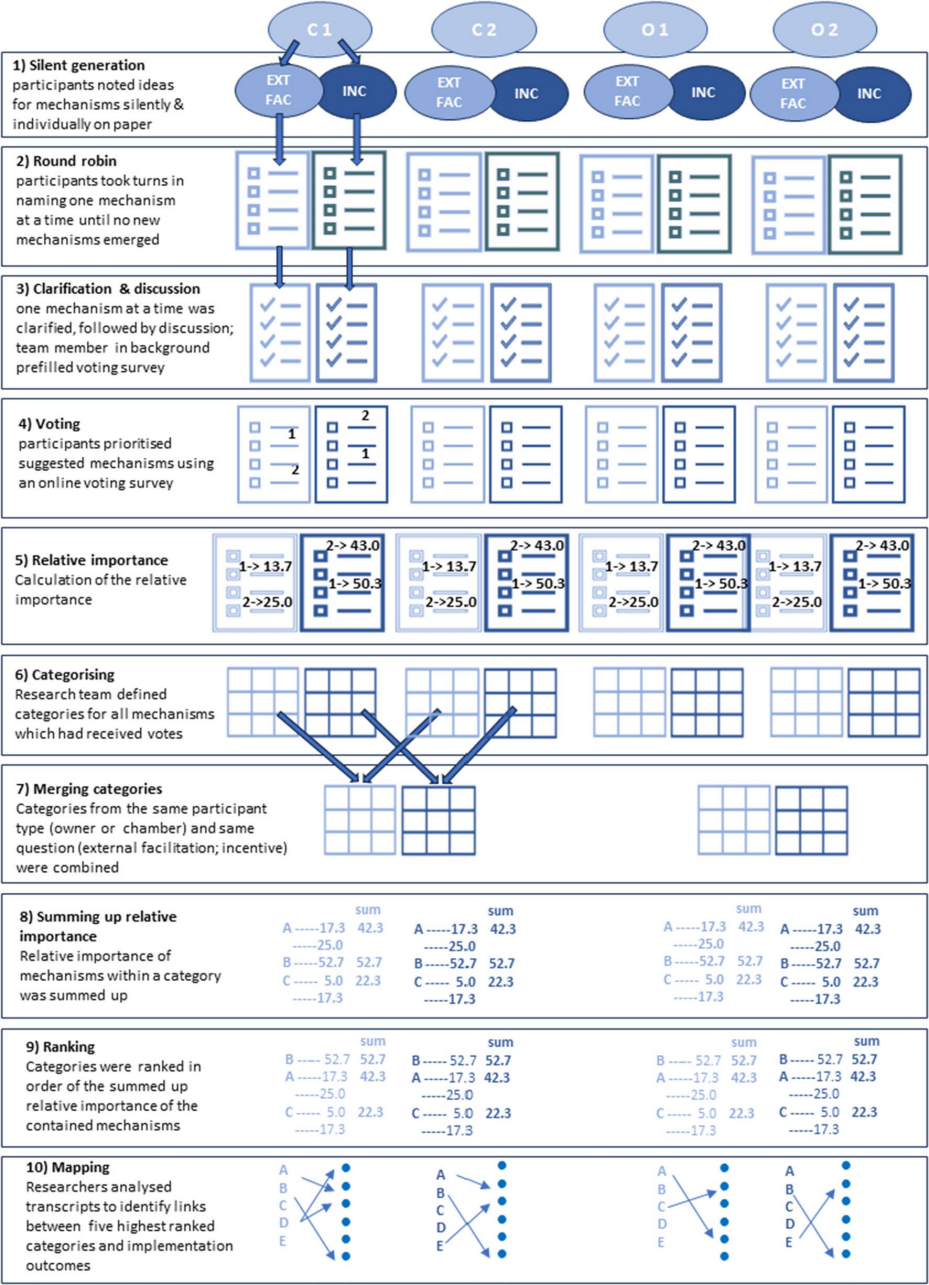
Illustration of the steps of the nominal group technique (NGT) and following analysis. C = group of chamber employees, O = group of owners, EXT FAC = external facilitation, INC = incentive, A,B,C,D,E = categories of mechanisms, ● = implementation outcomes

Figure 1 illustrates the approach taken. The NGT consisted of four steps: silent generation of ideas for mechanisms of change, round robin in which the participants shared their ideas one at a time, clarification and discussion of the suggested mechanisms, and voting for the potentially most effective mechanisms. Step 2 and 3 (round robin, clarification and discussion) were audio-recorded and transcribed ad verbatim.

The NGT-discussions were facilitated by the principal researcher (DM) with another experienced member of the research team also fluent in German (AEW or DD) following the discussion in the background. The principal researcher had prior experience with online NGT [33]. The other researchers noted the mechanisms which emerged in the discussion and populated the online voting tool to be used by participants in the final step of the NGT. Participants chose what they thought were the five most important mechanisms and ranked these by allocating 5 points to the most important and 1 point to the least important mechanism. All other mechanisms received no points.

### Quantitative analysis

All individual votes were summed per mechanism, frequencies of votes listed, and the relative importance (rI) calculated [40].

$$Relative\,importance\,\left(rI\right)=\frac{total\, score}{participants \, \times\, 15} \times 100$$

where *total score* is the sum of votes from all participants in a group, and *participants* is the number of participants in that group. Every participant had 5 + 4 + 3 + 2 + 1 = 15 points to allocate. Calculating the rI enables an overall ranking of mechanisms when NGT groups have different numbers of participants [40]. All mechanisms that received votes were taken forward for qualitative analysis.

### Qualitative analysis

Content analysis was applied to the German transcripts of the NGT-discussions by two researchers independently to define categories for the suggested mechanisms and to map the mechanisms to implementation outcomes [41, 42]. Grouping mechanisms into categories allowed summarising information and analysis across several NGT groups [43]. Similar and complementary mechanisms (e.g., “tutor for MR check in background” and “contact person for peer support”) from both sessions with the same participant type (i.e., pharmacy owners or chamber employees) were grouped together into a single category (e.g., “expert support line”). Any discrepancies were resolved by discussion within the research team. Resulting categories were ranked by adding up the rI of all mechanisms in that category. The research team then used the context of the clarification and discussion step to link the five highest ranked categories to potential implementation outcomes [44]. This study used Proctor’s set of implementation outcomes of *acceptability, adoption, appropriateness, cost, feasibility, fidelity, penetration and sustainability* [44, 45]. “*Acceptability*” captured whether the delivery of MRs in the community pharmacy setting in general was acceptable. “*Appropriateness*” referred to the perceived fit or compatibility of the MR service for a given practice setting. To illustrate the findings in a visual manner, all identified mechanisms of change and their links to implementation outcomes were organised in an adapted model of Smith’s suggestion for an Implementation Research Logic Model [20].

## Results

Twenty-eight key stakeholders replied to the recruitment survey. These included 17 pharmacy owners from 9 different pharmacy chambers and 11 chamber representatives from 8 different pharmacy chambers respectively. Four NGT-discussions were held in total (n = 2 owners; n = 2 chamber employees). The online NGT-discussions were held in early 2023 and lasted between 100 and 120 min.

**Fig. 2:**
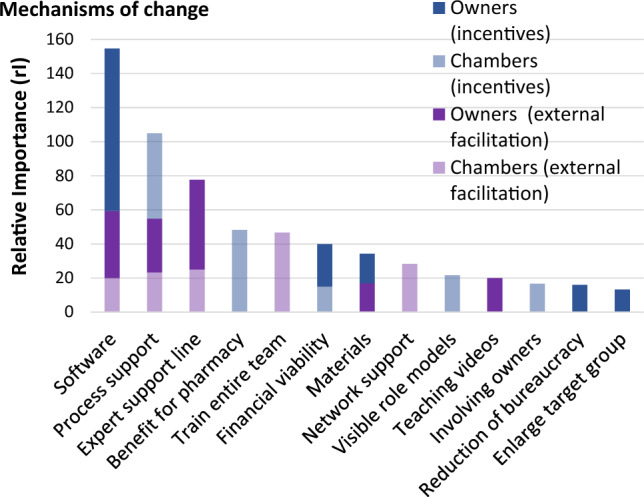
Relative importance (rI) of the five highest ranked categories of identified mechanisms as ranked per group (chamber employees; owners) and strategy (external facilitation; incentives)

Chamber employees generated 38 different mechanisms for “external facilitation” and 23 for “incentives”. Pharmacy owners generated 31 mechanisms for “external facilitation” and 19 for “incentives”. [Supplement 1] These were grouped into 24 distinct categories for “external facilitation” and 18 for “incentives” across both participant types. Table 2 displays the five highest ranked categories for “external facilitation” and Table 3 the five highest for “incentives” per participant group. Highest ranked mechanisms were software (rI = 154.7; linked to 4 implementation outcomes), process support (rI = 104.9), and an expert support line (rI = 77.7) which were linked to 3 implementation outcomes each. Software and process support were ranked highly both as mechanisms for external facilitation and as incentives. Most mechanisms of change were linked to 2 outcomes, however for “teaching videos” and “enlarge target group” no links to outcomes were mentioned. Figure 2 illustrates the rI for each distinct category of identified mechanisms of change.

**Table 2:** Five highest ranked categories for “external facilitation” with corresponding mechanisms

Category mechanism	Sum of votes	Frequency of votes	Relative importance*	Summed up relative importance	Linked to implementation outcomes
*Chamber employees*					
**Train entire pharmacy team**				46.7	Appropriateness, feasibility, penetration
Providing information to technicians and other professionals on how to recruit patients (C2)	12	4	20.0		
Coaching/teaching the entire pharmacy team (C2)	10	3	16.7		
Educating pharmacy team on organisational procedures (C2)	4	2	6.7		
Organising education sessions for all professional groups working at the pharmacy (C1)	1	1	1.7		
Providing a coach as a facilitator between experienced colleagues and pre-registration pharmacists (C2)	1	1	1.7		
**Network support**				28.3	Appropriateness, feasibility
Facilitating networking (intersectional/ interprofessional) (C1)	6	2	10.0		
Shadowing activities in experts' pharmacies (C2)	6	2	10.0		
Helping pharmacists to help themselves/spread word about MR databases (C1)	4	1	6.7		
Supporting educational sessions/seminars for pharmacists and doctors (C2)	1	1	1.7		
**Expert support line**				25.0	Feasibility
Setting up a support centre (C2)	15	3	25.0		
**Process support**				23.3	Adoption, feasibility
Alleviating the pressure associated with the pharmaceutical daily routine (C1)	14	3	23.3		
**Software**				20.0	Feasibility
Automating the documentation of medications and MR in one single system (C1)	12	3	20.0		
*Owners*					
**Expert support line**				52.7	Adoption, fidelity
Providing a tutor (in background), who can review MRs for beginners (O1)	18	4	30.0		
Availability of an external contact person or peer support to answer pharmaceutical questions on request (O2)	17	5	22.7		
**Software**				39.4	Appropriateness, adoption, feasibility
Centralised and standardised software (O1)	10	3	16.7		
Providing a modular system or integration of MRs in pharmacy software (O2)	9	4	12.0		
Providing an MR online-tool with search-/ filter function (O2)	8	2	10.7		
**Process support**				31.6	Appropriateness, adoption, feasibility
Providing external support with MR time management and help scheduling (O1)	12	3	20.0		
Repeating workshops covering the detailed steps of an MR (O1)	3	1	5.0		
Providing support for all steps of the MR-process (from provision, documentation to billing) (O1)	2	1	3.3		
Providing a template collection for process organisation (O1)	2	1	3.3		
**Teaching videos**				20.0	–
Providing educational videos to fill knowledge gaps (O2)	15	4	20.0		
**Materials**				17.0	Appropriateness, adoption, feasibility
Developing templates for adverts that target patient groups (O1)	3	1	5.0		
Developing ready-to-use materials that can be adapted (team & patients) (O2)	9	3	12.0

**Table 3:** Five highest ranked categories for “incentives” with corresponding mechanisms

Category mechanism	Sum of votes	Frequency of votes	Relative importance*	Summed up relative importance	Linked to implementation outcomes
*Chamber employees*					
**Process support**				50.0	Adoption, feasibility
Supporting the infrastructural development of MRs (incl. initial phase) (C1)	20	4	33.3		
Providing pre-phrased process descriptions as part of the quality management process (C1)	10	4	16.7		
**Benefit for pharmacy**				48.3	Sustainability
Inspiring young colleagues to take up community pharmacy (C1)	8	3	13.3		
Increasing employees' job-satisfaction (C1)	7	2	11.7		
Stressing advantages of MRs and to owners e.g., staff loyalty (C2)	6	2	10.0		
Facilitating recruitment of junior staff (C2)	2	2	3.3		
Generating more profit using specialist knowledge (C2)	3	1	5.0		
Potential of MR related work to be carried out at home (C2)	3	1	5.0		
**Increase visibility of role models**				21.7	Appropriateness, adoption
Making role models visible (e.g., via social media posts, newsletters) (C2)	13	3	21.7		
**Involving owners**				16.7	Appropriateness, adoption
Finding ways of reaching owners (C2)	10	2	16.7		
**Financial viability**				15.0	Cost
Reducing costs of materials (C1)	9	3	15.0		
**Owners**					
**Software**				95.3	Appropriateness, adoption, fidelity
Integrating MR software within pharmacy management software (O1)	15	4	25.0		
Providing maximal IT support for the entire process (O1)	15	4	25.0		
Providing access to fit-for-purpose software (O2)	16	4	21.3		
Providing easy to use appointment software (clever & simple) (O2)	8	3	10.7		
Providing simple, intuitive usable software (O2)	10	2	13.3		
**Financial viability**				25.0	Adoption, cost
Offering higher remuneration during the initial phase (external incentive) (O1)	15	4	25.0		
**Materials**				17.3	Appropriateness
Providing a start-up package (materials) (O2)	13	4	17.3		
**Reduction of bureaucracy**				16.0	Appropriateness, adoption, feasibility
Reducing bureaucracy and documentation (O2)	7	3	9.3		
Simplifying customers' input into documentation (O2)	5	2	6.7		
**Larger target group**				13.3	–
Establishing the legal base to provide MRs for patients in care homes (O1)	8	3	13.3

Both chamber employees (C) and pharmacy owners (O) called for fit-for-purpose software which would ideally be integrated into the existing pharmacy software, followed a standardised layout, and had a broad database with search and filter function for frequent medication problems.

“To simply reduce the time lost by manual registering and data editing. In the end, the doctor’s letter and literature that needs to be sent on, should be sent from the existing pharmacy software, not a separate one.” (Chamber employee, C12).

A fit-for-purpose software was perceived by owners by far as the most important incentive to increase MR adoption and feasibility.

“Well, for me it would be the biggest incentive, a very simple [software] tool that can be used intuitively.” (Owner, O23).

An expert support line for clinical feedback was ranked highly by both chamber employees and pharmacy owners. Owners stressed that having an expert available in the background was important in making the decision to adopt the MR service in their pharmacies and to ensure fidelity.

“We were not quite sure if we had got [the MR] right. [We appreciate] the opportunity to send it to a tutor who double checks, and then confirms or adds another point.” (O12).

Chamber employees highlighted that a support line would also make MRs more feasible since the time to double check a medication review assessment was often unavailable. Process support included suggestions by owners for standard operating procedures (SOP), re-developed roles for pharmacists and technicians, and.

“identification and correct placement of time resources [of individuals] to ensure no single staff member nor the entire team becomes overwhelmed.” (O14).

Chamber employees deemed process support as the most effective incentive to encourage pharmacists to adopt MRs.

“[I mean], that an external person visits the pharmacy to aid the development of correct structures […] and also supports the first MRs.” (C13).

Chamber employees perceived training the entire pharmacy team a highly important mechanism to improve feasibility and penetration.

“I believe it is very important to include technicians because they are always at the counter and have the most contact with patients.” (C22).

Training was also deemed crucial to facilitate the adoption of MRs: “Do technicians feel addressed [in seminars]? Are they aware of the service? … Which [patients] can they refer to the pharmacist?” (C24).

According to chamber employees suitable network support to connect pharmacists with each other and with other health care professionals would contribute to the appropriateness and feasibility of MRs.

“I was thinking of a best practice approach. That experienced colleagues visit other pharmacies or invite [beginners] to show them how it’s done.” (C22).

Teaching videos and development of templates were deemed important by pharmacy owners to fill knowledge gaps of team members, in particular adaptable templates for patient information leaflets about the MR service. Owners explained that materials, more specifically a starter kit containing recruitment flyers, SOPs, checklists for the pharmaceutical assessment and instructions for billing would be a powerful incentive and contribute to MRs’ appropriateness, adoption, and feasibility in their pharmacies.

A range of benefits for pharmacies to incentivise pharmacists to implement MRs were proposed by chamber employees.

“The incentive could be … the emphasis on the uniqueness of the pharmacy profession. Not … the easier-to-provide logistic services that anyone else can provide.” (C23).

Implementing MRs would in turn increase pharmacists’ job satisfaction and thus lead to higher staff loyalty. Moreover, noticeable benefits for pharmacists would in the end facilitate recruitment of future staff and hence contribute to the sustainability of MR delivery. Chamber employees discussed the visibility of positive role models for owners and thought this could be a powerful incentive:

“To feature someone who performs MRs as a role model, …who says I love my job … so that others can see that it works.” (C23).

Chamber employees stressed the importance of reaching and involving pharmacy owners in the implementation process. They considered involving owners an important incentive for pharmacy teams which would improve appropriateness and adoption of MRs.

“We are struggling to reach those owners who need a jolt. You’d probably have to use several channels to finally get there.” (C23).

Owners suggested improving the financial viability by paying a higher remuneration in the initial phase as.

“[the MR] is calculated to last 90 min… and that’s not feasible in the beginning.” (O11) and therefore a “start-up financing” (O13) should be offered.

In addition, a substantial reduction of bureaucracy would add to appropriateness, adoption, and feasibility of MR-implementation according to owners. This included suggestions for less documentation of other pharmacy tasks but also the simplification of the over complicated bureaucracy around MRs.

“An hour’s patient interview shouldn’t be followed by half an hour of documentation.” (O22).

Finally, clarifying the regulations to include patients who were living in care homes (larger target group), would incentivise some owners as they felt that MRs were particularly important for that patient group.

**Fig. 3:**
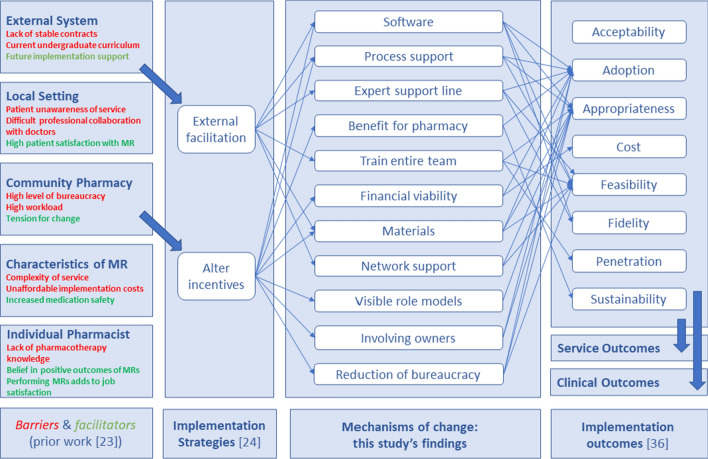
A suggested implementation model underpinned by theory

The five highest ranked mechanisms per group (chamber employees, pharmacy owners) and question (*external facilitation, incentives*) were included in a model for implementing MRs in community pharmacies [Fig. 3]. The links to implementation outcomes in this model were based on the context and explanations which participants had given. The first and second column of the illustration are populated with findings from a prior study of this research group which identified barriers and facilitators as well as potential implementation strategies [28]. The third column depicts the mechanisms of change as prioritised by this study’s participants. Thin arrows present links between mechanisms of change and the respective implementation outcomes (far-right column) which were made by participants.

## Discussion

### Key findings

This study presents key stakeholders' priorities for mechanisms of change which are suited to inform a nationwide implementation plan. Among the highest ranked mechanisms were fit-for-purpose software, process support detailing all steps of the MR, and an expert support line for pharmacotherapeutic questions that could be contacted as required. These three mechanisms along with financial viability were prioritised by both groups of participants. Further suggestions for external facilitation included training the entire pharmacy team, and support of networking so that implementing pharmacists could learn from each other. Owners stated that putting into practice the mechanisms within the category “external facilitation” would incentivise them to implement MRs, in addition they suggested a start-up financing. Chamber employees prioritised rather intangible incentives such as higher job satisfaction and easier staff recruitment which would benefit MR-providing pharmacies in the long term.

### Strengths and weaknesses

A strength of this study is that it involved two types of key decision makers: pharmacy owners, and the organisations which shape the external support. Besides, the study followed the Implementation Research Logic Model (IRLM) and is rigorously underpinned by implementation theory, namely the Framework for Implementing Services in Pharmacy [46], Expert Recommendations for Implementing Change (ERIC) [29] and Proctor’s taxonomy for implementation outcomes [44] as illustrated in Fig. 2. A limitation is the small number of participants. However, in the second round of discussions, data saturation was achieved. Another limitation is that as participation was voluntary, it is possible that participants who had an interest in the MR service, were more likely to sign up for the study. In addition, participants from chambers might have focused on mechanisms already in use or in concise planning than on completely new ones. In contrast, pharmacy owners might have focused on mechanisms not (yet) in use in their respective pharmacies. Lastly, excluding pharmacist employees, who are the active care providers, from the NGT can also be seen as a limitation as it can be argued that suggestions for change often work better from a bottom up rather than a top-down approach.

### Interpretation

Based on the Exploration Preparation Implementation Sustainment framework (EPIS) [47], the German community pharmacy system is currently at an early exploration and preparation stage of implementation of MRs [11]. This is evidenced by the fact that stakeholders in this study were focusing on getting started rather than being concerned with the quality of MR-delivery (*fidelity*), performing large numbers of MRs (*penetration*) or keeping up with delivery (*sustainability*). Consequently, many of the suggested mechanisms targeted the early implementation outcomes *appropriateness*, *adoption* and *feasibility* [Fig. 2], and this focus is highly recommended and expected at the start of every implementation process [45]. None of the participants mentioned any mechanisms to improve acceptability of MRs suggesting a basic agreement of MRs’ *acceptability*. This finding indicates that the very first step towards implementation, convincing key stakeholders of MRs’ value, has been achieved.

Some of the mechanisms identified in this study are similar to those in the literature [19, 48], however it was important to ascertain stakeholders’ priorities as resources for implementation are limited and efforts need to be focused. Fit-for-purpose software was ranked by far highest across both owners and chamber employees and was the only mechanism to target 4 different implementation outcomes: *adoption, appropriateness, feasibility, and fidelity* mirroring other published studies [48, 49]. Well-designed software for documentation and patient identification was amongst the most important mechanisms identified by MacKeigan et al. who investigated implementation of an integrated MR-programme in Canadian community pharmacies [48].

Training the entire pharmacy team was linked to *appropriateness* and *feasibility* but also perceived to target the mid to long-term outcome *penetration.* This aligns well with the literature as including technicians in some MR-tasks has been shown to improve efficiency of MR-delivery and thus reach more patients [19, 48–53]. Several studies stressed the importance of engaging pharmacy managers in the implementation process and concluded that connecting managers with each other to either shadow others or to be coached in their own pharmacy was successful which makes our participants’ suggestion to involve owners and support networking promising mechanisms [48, 53, 54]. This study’s participants agreed that process support for all steps of an MR and an expert support line were highly important. This is in line with Stafford et al. who illustrated that employing these mechanisms improved implementation outcomes over time [53]. Varas Doval et al. used detailed process support provided by practice change facilitators who visited the pharmacies, and obliged owners to attend a half-day information session which resulted in 55% of participating pharmacies implementing MRs despite no available remuneration [54]. Chamber employees proposed a subsidy on materials or software to reduce implementation costs and owners suggested a start-up financing to incentivise adoption of MRs. The negotiated remuneration of 90€ per MR was calculated in 2021 to cover 80 min of a pharmacist’s time. However, wages and other costs have continuously risen since then. In addition, a certain level of experience is necessary to deliver all steps of an MR within the allocated time frame. In view of this, a start-up financing appears to be reasonable as an implementation study had quantified implementation costs at 28,000€ (Spain, in 2017) for the initial implementation of MRs in one pharmacy [55]. Otherwise, some pharmacy owners might be tempted to follow the example of UK pharmacy managers who focused solely on the financial side when implementing the medicines use review (MUR, a simpler version of an MR) [56]. Many UK pharmacy chains initially set target numbers to be met by their staff in order to facilitate implementation [57]. However, this strategy resulted in large quantities of poor quality MURs [58], and eventually led to the service being decommissioned by the UK health authorities [59].

### Further research

Implementing new services requires an array of implementation strategies at various levels. It is crucial to involve stakeholders of a given setting in determining which mechanisms of change are likely to realise the strategies and to improve implementation outcomes. Therefore, it would be beneficial to include additional stakeholders, such as employed pharmacists (of different ages, levels of experience) and patients in future research. A hybrid implementation study which measures both implementation and clinical outcomes appears to be a promising way to determine the practical value of the here identified and prioritised mechanisms of change [60].

## Conclusion

This study identified key stakeholders’ priorities for mechanisms of change to implement medication reviews in the community pharmacy setting. Focusing implementation efforts on the prioritised mechanisms (e.g., fit-for purpose software, detailed process support, expert support line, training the entire pharmacy team, support network building) is likely to significantly progress a national implementation plan for community pharmacies in Germany and other countries which are currently at an early implementation stage.

### Supplementary Information

Below is the link to the electronic supplementary material.

Supplementary file1 (DOCX 57 kb)
