# Supplementary material for: Implementation of medication reviews in community pharmacy: reaching consensus on stakeholders’ recommendations for mechanisms of change using the nominal group technique
Source: Int J Clin Pharm. 2024 Mar 15;46(3):714–26. doi: 10.1007/s11096-024-01708-y (PMC11133082; doi:10.1007/s11096-024-01708-y)
Supplement: Supplementary file 1 — Supplementary file1 (DOCX 57 kb) [file 11096_2024_1708_MOESM1_ESM.docx]

**NGT step 4:** All mechanisms of change that were named for the final voting step, sorted by question (external facilitation / incentives) and participant group (Chambers / pharmacy owners)

| **CHAMBERS 1 -EXTERNAL FACILITATION** |  |  |  |  | **SUM** | **FREQUENCY** |
| --- | --- | --- | --- | --- | --- | --- |
| Relief for pharmaceutical daily routine (staff / logistics) | 4 |  | 5 | 5 | 14 | 3 |
| Automation of medication and documentation in one system | 5 | 3 | 4 |  | 12 | 3 |
| Establishing a pool of experts / coaches |  | 5 |  | 2 | 7 | 2 |
| Facilitate networking (intersectional / interprofessional) |  | 2 |  | 4 | 6 | 2 |
| Address staff shortage through mentorships (all professional groups) | 3 |  | 2 |  | 5 | 2 |
| Connection to KIM (existing safe communication system for all health professionals) |  | 1 | 1 |  | 2 | 2 |
| Help pharmacists to help themselves / spread word about MR databases |  | 4 |  |  | 4 | 1 |
| Externals manage the communication with prescribers |  |  | 3 |  | 3 | 1 |
| Online trainings (on demand) |  |  |  | 3 | 3 | 1 |
| Voluntary quality check (like online exams) (Ringversuch) in initial phase of providing MRs | 2 |  |  |  | 2 | 1 |
| Education sessions for all professional groups |  |  |  | 1 | 1 | 1 |
| List of benefits to be used when recruiting patients | 1 |  |  |  | 1 | 1 |
| Share positive experiences |  |  |  |  |  |  |
| Central production of informative videoclips / flyers |  |  |  |  |  |  |
| Expert opinion on pharmaceutical problems (via email) |  |  |  |  |  |  |
| Individualised, demand-oriented, interactive training |  |  |  |  |  |  |
| Prepared information for prescribers to present benefit and scope of MRs |  |  |  |  |  |  |
| Expand scope of medication information service (at chambers) |  |  |  |  |  |  |
| Expand teaching of MRs for students in their pre-registration year (workshop/ case training) |  |  |  |  |  |  |
| Shadowing at experts' pharmacies |  |  |  |  |  |  |
| Mystery shopper for organisational process |  |  |  |  |  |  |
| Gamification of knowledge (1000-piece jigsaw) |  |  |  |  |  |  |

| **CHAMBERS 2 – EXTERNAL FACILITATION** |  |  |  |  | **SUM** | **FREQUENCY** |
| --- | --- | --- | --- | --- | --- | --- |
| Information for technicians and other professionals how to recruit patients | 1 | 4 | 4 | 3 | 12 | 4 |
| Support centre | 2 | 3 | 5 | 5 | 15 | 3 |
| Coaching / teaching the entire team | 4 | 2 |  | 4 | 10 | 3 |
| Support shadowing at experts' pharmacies | 5 |  |  | 1 | 6 | 2 |
| Educating team on organisational procedures |  |  | 2 | 2 | 4 | 2 |
| Diary on successful MRs |  | 5 |  |  | 5 | 1 |
| Case study training including individual feedback |  |  | 3 |  | 3 | 1 |
| "CIRS" for MRs (critical incident reporting system) | 3 |  |  |  | 3 | 1 |
| Supported education session / seminar for pharmacists and prescribers |  |  | 1 |  | 1 | 1 |
| Coach as bridge between seasoned colleagues and pre-registration pharmacists |  | 1 |  |  | 1 | 1 |
| Training on prioritisation of DRPs |  |  |  |  |  |  |
| SOP for organisational procedures and implementation of MRs |  |  |  |  |  |  |
| Collection of practice cases and questions to be used in professional development seminars / workshops |  |  |  |  |  |  |
| Motivational programme for young pharmacists |  |  |  |  |  |  |
| Presentation and assessment of everyday support materials |  |  |  |  |  |  |
| Compilation and teaching of Helpful phrases to be used in dialogue with patients and prescribers |  |  |  |  |  |  |

| **OWNERS 1 - EXTERNAL FACILITATION** |  |  |  |  | **SUM** | **FREQUENCY** |
| --- | --- | --- | --- | --- | --- | --- |
| Tutor (in background), who can check MRs for beginners | 5 | 5 | 3 | 5 | 18 | 4 |
| External organisational support to detect time resources and helps scheduling | 2 |  | 4 | 2 | 12 | 3 |
| Superordinated central and standardised software | 1 |  | 5 | 4 | 10 | 3 |
| Training of communication with prescribers and patients | 3 | 1 |  |  | 4 | 2 |
| Support to focus on relevant (drug related) problems |  | 4 |  |  | 4 | 1 |
| Supervisor, who accompanies first couple of MRs | 4 |  |  |  | 4 | 1 |
| Recurring workshops on the detailed steps of an MRs |  | 3 |  |  | 3 | 1 |
| Template for adverts that target patient groups |  |  |  | 3 | 3 | 1 |
| Support for all steps of the MR-process (from provision, documentation to billing) |  | 2 |  |  | 2 | 1 |
| Template collection for process organisation |  |  | 2 |  | 2 | 1 |
| Determine training needs around materials (entire team) |  |  | 1 |  | 1 | 1 |
| A forum / platform for everyday problems (with suggestions for solutions) |  |  |  | 1 | 1 | 1 |
| Economic calculations for individual pharmacies |  |  |  |  |  |  |
| Team education on how to identify eligible patients |  |  |  |  |  |  |
| Team education on the purpose and aim of MRs |  |  |  |  |  |  |
| Operational plan for task distribution within the team |  |  |  |  |  |  |
| Suggestions for solutions |  |  |  |  |  |  |
| ABDA (Federal Union of German Association of Pharmacists) field service to increase prescribers' acceptance of MRs |  |  |  |  |  |  |

| **OWNERS 2 EXTERNAL FACILITATION** |  |  |  |  |  | **SUM** | **FREQUENCY** |
| --- | --- | --- | --- | --- | --- | --- | --- |
| Contact person / Peer support on request (extern) | 3 | 5 | 2 | 4 | 3 | 17 | 5 |
| Educational videos to fill knowledge gaps | 5 | 2 | 5 | 3 |  | 15 | 4 |
| Modular system / integration in pharmacy software | 2 |  | 1 | 5 | 1 | 9 | 4 |
| Online pool with example MRs | 1 | 3 |  | 1 | 2 | 7 | 4 |
| Ready-to-use materials that can be adapted (team & patients) | 4 |  | 3 | 2 |  | 9 | 3 |
| MR online-tool with search-/ filter function |  |  | 4 |  | 4 | 8 | 2 |
| Temporary subsidy for software tools |  | 1 |  |  | 5 | 6 | 2 |
| Raising awareness in team for stronger patient loyalty (that can be reached by providing MRs) |  | 4 |  |  |  | 4 | 1 |
| Location analysis (in direct setting) |  |  |  |  |  |  |  |
| SOPs (e.g. the criteria for patient recruitment ) |  |  |  |  |  |  |  |
| Efficiency via digitalisation |  |  |  |  |  |  |  |
| Positive dialogue transition (from dispensing to offering the service) |  |  |  |  |  |  |  |
| Positive presentation towards prescribers (building blocks of good communication) |  |  |  |  |  |  |  |

| **CHAMBER 1 INCENTIVES** |  |  |  |  | **SUM** | **FREQUENCY** |
| --- | --- | --- | --- | --- | --- | --- |
| Process support for MRs (incl. initial phase) | 5 | 5 | 5 | 5 | 20 | 4 |
| Ready-to-use SOPs for quality management process | 3 | 2 | 1 | 4 | 10 | 4 |
| Reduced costs of materials | 4 |  | 4 | 1 | 9 | 3 |
| Inspire and recruit young colleagues | 2 | 3 | 3 |  | 8 | 3 |
| Experience exchange groups for all team members | 1 |  | 2 | 2 | 5 | 3 |
| Increase employees' job-satisfaction (employees' loyalty to pharmacy) |  | 4 |  | 3 | 7 | 2 |
| Challenge |  | 1 |  |  | 1 | 1 |

| **CHAMBER 2 INCENTIVES** |  |  |  |  | **SUM** | **FREQUENCY** |
| --- | --- | --- | --- | --- | --- | --- |
| Make role models visible | 5 | 4 | 4 |  | 13 | 3 |
| Centralised publicity to increase patient demand | 1 |  | 2 | 4 | 7 | 3 |
| Find channels to reach owners |  | 5 | 5 |  | 10 | 2 |
| Stressing advantages e.g., staff loyalty |  |  | 3 | 3 | 6 | 2 |
| Neatly designed packages with announcement and readily available campaigning material | 4 |  |  | 1 | 5 | 2 |
| Simplified documentation for MR |  | 2 |  | 2 | 4 | 2 |
| Highlight easier recruitment of junior staff |  | 1 | 1 |  | 2 | 2 |
| Incentives to get prescribers on board |  |  |  | 5 | 5 | 1 |
| Use specialist knowledge as a strategy to generate more profit |  | 3 |  |  | 3 | 1 |
| Enable MR related work as home office task | 3 |  |  |  | 3 | 1 |
| MR contest in between pharmacies (intra & inter) | 2 |  |  |  | 2 | 1 |
| Financial start-up support |  |  |  |  |  |  |
| Offer continuing education within working hours |  |  |  |  |  |  |
| Publication of MR-providing pharmacies by independent bodies |  |  |  |  |  |  |
| Use of health campaign days (e.g., world asthma day, blood pressure) to promote local advertisement |  |  |  |  |  |  |
| Use of health campaign days (e.g., world asthma day, blood pressure) to promote local advertisement |  |  |  |  |  |  |

| **OWNERS 1 INCENTIVES** | | | | | **SUM** | **FREQUENCY** |
| --- | --- | --- | --- | --- | --- | --- |
| Integration in pharmacy management software | 5 | 5 | 3 | 2 | 15 | 4 |
| Higher remuneration during the initial phase (external incentive) | 2 | 4 | 4 | 5 | 15 | 4 |
| Maximal IT support for the entire process | 3 | 3 | 5 | 4 | 15 | 4 |
| Legal base to provide MRs for patients in care homes | 4 | 1 |  | 3 | 8 | 3 |
| Team Challenge (incentive in inner setting) | 1 | 2 | 2 |  | 5 | 3 |
| Quality certification for pharmacies (including advertisements) |  |  | 1 | 1 | 2 | 2 |
| Increase of pharmacies' appeal as a workplace |  |  |  |  |  |  |

| **OWNERS 2 Incentives** |  |  |  |  |  | **Sum** | **Frequency** |
| --- | --- | --- | --- | --- | --- | --- | --- |
| Good Software | 5 |  | 1 | 5 | 5 | 16 | 4 |
| Start-up package (materials) | 4 | 4 |  | 4 | 1 | 13 | 4 |
| Easy to use appointment software (clever & simple) | 3 | 1 | 4 |  |  | 8 | 3 |
| Reduction of bureaucracy and documentation |  | 2 |  | 1 | 4 | 7 | 3 |
| MR referrals by prescribers |  | 3 | 2 |  | 2 | 7 | 3 |
| Simple, intuitive use of software |  | 5 | 5 |  |  | 10 | 2 |
| Visibility in "pharmacy manager" (official public online-list of pharmacies) |  |  | 3 | 2 |  | 5 | 2 |
| Customers' value for 2 signatures | 2 |  |  |  | 3 | 5 | 2 |
| Positive contribution margin |  |  |  | 3 |  | 3 | 1 |
| Additional bonus (e.g., 20% share + paid overtime hours) | 1 |  |  |  |  | 1 | 1 |
| Free database access (for a limited time span) |  |  |  |  |  |  |  |
| Outcome agreement for all team members |  |  |  |  |  |  |  |
